# Incidental Papillary Microcarcinoma and Papillary Thyroid Carcinoma in Multinodular Goiter

**DOI:** 10.1155/2023/2768344

**Published:** 2023-01-14

**Authors:** Cigdem D. Arican, Tulin Ozturk, Muhammet Sait Sager, Ipek Sertbudak, Serkan Teksoz, Cansu Turker Saricoban, Abdulkerim Uygur

**Affiliations:** ^1^Pathology Department, Sancaktepe Sehit Prof. Dr. Ilhan Varank Sancaktepe Training and Research Hospital, Istanbul 34785, Turkey; ^2^Pathology Department, Istanbul University Cerrahpasa Medical Faculty, Istanbul 34096, Turkey; ^3^Nuclear Medicine Department, Istanbul University Cerrahpasa Medical Faculty, Istanbul 34096, Turkey; ^4^General Surgery Department, Istanbul University Cerrahpasa Medical Faculty, Istanbul 34096, Turkey; ^5^Public Health Department, Istanbul University Cerrahpasa Medical Faculty, Istanbul 34096, Turkey

## Abstract

**Introduction:**

This study aimed to examine the incidence of incidental papillary microcarcinoma (PMC) and papillary thyroid carcinoma (PTC) in patients with benign multinodular goiter (MNG) and to compare their relationship with some prognostic factors from a new perspective.

**Methods:**

Bilateral total thyroidectomy (BTT) was used to evaluate the data of 716 patients who underwent a surgery for MNG. The prognostic data for these tumors and the relationship between patients with bilateral and multifocal tumors were evaluated using statistical tests.

**Results:**

Papillary carcinomas were detected in 201 patients, PMC in 134 of them, and PTCs in 67. Bilaterality was more common in patients with PTCs than in those with PMC. The incidence of bilaterality in male patients with PTC was statistically more common. The presence of intra-tumoral lymphocytes was higher in multifocal PTC cases than in unifocal PTC cases.

**Conclusion:**

The results revealed that the number of PMC s was high in incidental tumors, and patients with PTC with male sex, bilaterality, multifocality, and tumor capsule invasion were associated with poor prognosis.

## 1. Introduction

Papillary thyroid carcinoma (PTC) is the most common type of thyroid cancer [1–3]. PTCs with a tumor diameter less than or equal to 1 cm are known as papillary microcarcinomas (PMC) [2, 4]. PMCs are commonly discovered during a detailed Ultrasonography (USG) examination of the neck for another reason or during histopathologic evaluation after thyroidectomy for multinodular goiter (MNG). The incidence of PMC in autopsy series has been reported as 36% [5]. In one study, the increased incidence of PTC seemed to cause these tumors as the third most common tumor in women [6]. Our study investigated the incidence of incidentally detected PMCs and PTCs in patients with MNG. The prognostic data of these tumors and the differences between patients with bilateral and multifocal tumors were also examined. These results were compared with those reported in the literature.

## 2. Materials and Methods

In this study, the pathology results of Bilateral Total Thyroidectomy (BTT) materials from 716 patients diagnosed with MNG at the Istanbul University—Cerrahpasa Faculty of Medicine between 2016 and 2021 were evaluated retrospectively. The incidence of PMC and PTC, as well as age, sex, bilaterality, multifocality, and intra- or extra-tumoral lymphocytic infiltration/fibrosis, vascular invasion (VI), and tumor capsule invasion (TCI) were examined. In addition, a comparison was made between patients with bilateral and multifocal PMC and those with PTC. Patients with a fine-needle aspiration biopsy (FNAB) result of as malignant, suspicious for malignancy, or atypia of undetermined significance were not included in this study. The R software (version 4.0.5) was used for statistical analyses (R: A language and environment for statistical computing. R Foundation for Statistical Computing, Vienna, Austria, available online: http://www.r-project.org/). The clinical and pathological features of the tumors except tumor size are presented as frequencies (percentage) and were assessed using chi-square test and Fisher's exact test. Only tumor size was shown as median (25th–75th percentiles) and analyzed using the Mann–Whitney *U* test. Statistical significance was set at *p* < 0.05. The authors confirm that there is complete anonymity with the data and the findings, and that ethical approval was obtained from the Sancaktepe Research and Training Hospital Scientific Research and Ethics Committee in 16.02.2022 (No. E-46059653-020) for this research.

## 3. Results and Discussion

Out of 716 patients, 60% (*n* = 428) were over 45 years of age, 76% were female (*n* = 543), and 24% were male (*n* = 173). The results showed that 65% of the patients had adenomatous and diffuse hyperplasia (ADH; *n* = 464), PMC 19% (*n* = 134), PTC in 9% (*n* = 67), and other tumors 7% [follicular adenoma (FA), follicular carcinoma (FC), medullary carcinoma (MC), oncocytic cell adenoma (OCA), and oncocytic cell carcinoma (OCC)] in Table 1.

**Figure 1:**
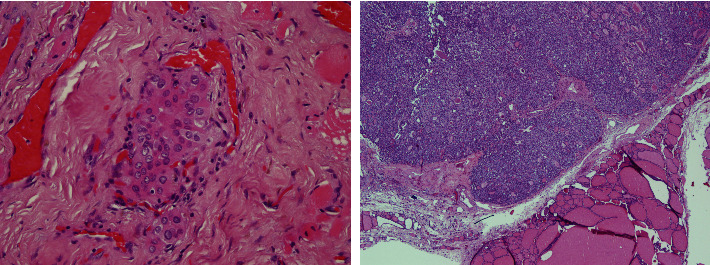
On the right, vascular invasion is seen in the case of PTC observed in the sections prepared by Hematoxylin & Eosin staining (H&E × 100), whereas on the left, capsular invasion is observed in the case of PTC observed in H&E sections (H&E × 100).

The rate of bilaterality was higher in PTCs than in PMCs (30% vs. 17%). Patients with PTC were in advanced stages at a higher rate than those with PMCs (*p* < 0.001; Table 2). PTCs had more TCI and VI than PMCs did (Figure 1). There were no statistical differences in other parameters.

**Figure 2:**
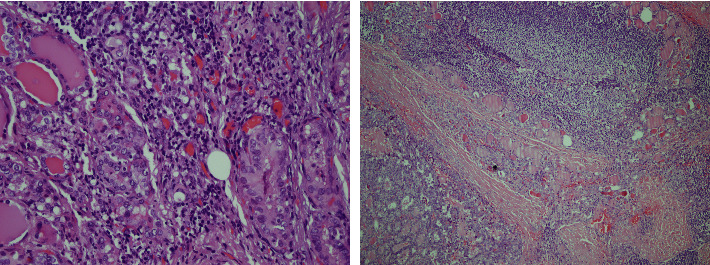
On the right, intra-tumoral lymphocyte infiltration is seen in the PMC case in Hematoxylin & Eosin sections (H&E × 400), whereas on the left, nontumoral lymphocyte infiltration is seen in the PTC case in H&E sections (H&E × 100).

Sixty-four percent (*n* = 86) of the 134 PMC patients were over 45, and the female/male ratio was 5/1. The tumor size ranged from 0.2 to 0.9 cm. Fifty-five percent of 67 PTC patients were over 45, and the female/male ratio was 2/1. The tumor size ranged from 1.5 to 3.2 cm. While intra-tumoral lymphocyte infiltration was found to be 9% in PTCs, it was 4.5% in PMCs. Nontumoral lymphocyte infiltration was a similar extent in both the tumors (Figure 2).

Gender distribution shows that male patients had more bilateral PTCs than unilateral PTCs (Table 3, *p* < 0.032). Intra-tumoral and nontumoral lymphocyte infiltration was slightly more common in bilateral PTCs, but the difference was not statistically significant (*p* > 0.353 and *p* > 0.226).

There was no significant difference between unifocal and multifocal masses with respect to the significant variables examined (Table 4). Although the numbers were small, intra-tumoral and nontumoral lymphocyte infiltration was more frequent in multifocal PTCs than in unifocal PTCs.

### 3.1. Prognostic Factors for PTCs and PMCs

Radiation, iodine deficiency, and genetics are commonly cited causes of thyroid cancer. The prognostic progression of incidental tumors should be investigated [7]. Although tumors ≤1 cm can lead to a good prognosis, the biological behavior of some tumors may increase the risk of malignancy. In our study, the prognostic factors for PTCs and PMCs were compared with different factors.

#### 3.1.1. Incidence of PMC and PTC

The effects of radiation on thyroid cancer were significant after the Chernobyl accident [8]. Incidental PTC has been detected in patients with nuclear power plants around them [9–11]. A previous study found that patients with MNG had incidence of incidental thyroid cancer [12]. Another study showed that 203 of 884 patients with MNG had PMC, and lymph node metastasis and multifocality lead to poor prognosis [13]. In the present study, 464 of the 716 patients with MNG had ADH. Incidental tumors were observed in 252 patients. There were 134 patients with PMCs, 67 with PTCs, 23 with FA, 18 with FC, 5 with OCA, 3 with OCC, and 2 with MC. The researchers found that 224 patients had incidental cancer, 134 PMC patients were in the first-line treatment, and FC, OCC, and MC were observed in patients. These were significant findings of this study.

#### 3.1.2. Gender

Male sex with PTC and PMC is a factor associated with poor prognosis [14–16]. In one study, male sex, extra-thyroidal spread, lymph node metastasis, and the presence of distant metastasis were associated with poor prognosis, and at least two of them were found to increase mortality [17]. In the present study, most of the patients with PMC and PTC were female. However, the proportion of males was higher in the bilateral PTC group than in the unilateral PTC group (*p* < 0.032). Of the 11 patients with advanced-stage PTC (pT3), 7 were males. In addition, 5 of the 11 male patients with advanced stage (pT3) had bilateral tumors. These results indicate that an excess of male PTC patients can lead to a poor prognosis despite the limited number of patients with pT3. Females outnumbered males with bilateral and multifocal PMCs. Four of the 5 patients with advanced-stage PMC (pT3) were female.

#### 3.1.3. Multifocality and Bilaterality

Male sex and multifocality are important for the prognosis of patients with PMC and under 45 years of age, and lymph node metastasis and multifocality are crucial for the prognosis of patients with PMC and over 45 years of age [18]. In a study, rearranged in transformation/PTCs gene rearrangements were investigated in PMC cases, and multifocal foci were shown as tumor foci with different genetic sequences [19]. Leni et al. showed that increased BRAF V600E mutation in PMC cases was associated with tumor recurrence and mortality [20]. It has been suggested in different studies that the BRAFV600E mutation may be associated with extra-thyroidal spread, multifocality, and advanced stage [21, 22]. However, the number of patients included in those studies was limited. Studies in which the number of patients is increased and postoperative long-term follow-ups are included may reveal different results.

Multifocality and lymph node metastasis cause local recurrence in patients with PTC [23]. The number of multifocal foci lead to poor prognosis in patients with PMC [24]. In the present study, bilaterality was more common in PTCs than in PMCs (30% vs. 17%). Multifocality was in 20 (15%) of 134 PMC patients and in 13 (19%) of 67 PTC patients. Eight (seven male and one female) of the PMC patients had bilaterality with multifocality. Six (four male and two female) of the PTC patients had bilaterality with multifocality. Intra-tumoral fibrosis was detected more frequently in unifocal PTCs than in multifocal PTCs, although the results were not highly significant. Only two patients with bilateral PTC patients had nontumoral fibrosis. These results indicate that male PTC patients with multifocal and bilateral tumors have a poor prognosis.

*(1) Lymphocytic Infiltration*. In previous studies, blood platelet/lymphocyte ratio (PLR) and red cell distribution width (RDW) ratios in benign and malignant thyroid cases were examined, and their relationship with early diagnosis and malignancy was investigated. In those studies, it was noted the increase of RDW and PLR was associated with malignant thyroid nodules [24–26]. Previous studies also investigated the relationship between Hashimoto's thyroiditis and PTC and PMC [27], the chronic lymphocytic thyroiditis and PTC and PMC [28]. Some other studies suggest that lymphocyte accumulations in and around the thyroid tumors are also seen as the body's defense against the tumors [29, 30]. Similarly, lymphocyte infiltration in or around the tumor in PTC patients is associated with a good prognosis and protects against tumor recurrence [31, 32].

In this study, the relationship between intra-tumoral and nontumoral lymphocyte infiltrations and stage in PTC and PMC tumors was compared. Intra-tumoral and nontumoral lymphocyte infiltration was more frequent in multifocal PTCs than in unifocal PTCs. Two of six PTC patients with lymphocyte infiltration were in tumor stage pT3, two in pT2, and two in pT1. Lymphocytic infiltration was observed in 20 patients with PMC, and all of whom were in the tumor stage pT1. Four of the PTC patients with lymphocytic infiltration were in tumor stages pT2 and pT3, which supports a poor prognosis.

#### 3.1.4. Age

In a study of PTC patients, tumor recurrence was more common in male aged ≥45 years [33]. Vasileiadis et al. identified TCI as an independent risk factor in PMC patients aged ≥45 years [34]. In the present study, 86 of 134 patients with PMC were over 45 years of age. Only six of them were in tumor stages pT2–pT3. Thirty-seven of the 67 patients with PTC were aged 45 years or older. Twenty-one patients had advanced stages (pT2–pT3). Among the PTC patients, the advanced stage of 21 patients aged 45 years and over had a poor prognosis. Half of the 20 patients with bilateral PTC patients were ≥45 years of age. These findings were consistent with those reported in the literature.

#### 3.1.5. Vascular/Capsular Invasion and Tumor Stage

After the Chernobyl accident, more VI, near and distant metastases were reported in thyroid tumors [35]. In PTC studies, VI has been correlated with poor prognosis and recurrence [36, 37]. In another study, age, multifocality, extensive metastasis, and TCI lead to poor prognosis in PMC cases [38]. In the present study, out of 20 patients with TCI, 5 had bilaterality, 3 multifocality, and 13 had advanced stages of pT2–pT3. In addition, VI was observed in 4 of the 67 PTC patients. All three of these tumors were classified as advanced-stage tumors (pT3). TCI was noted in 12 of 134 PMC patients, and 3 of these patients had bilaterality, 1 had multifocality, and 1 had a tumor stage of pT2. VI was not detected in the PMC tumors. PTC tumors appeared to have more TCI and were more likely to be at an advanced stage than PMC tumors. These results suggest that TCI and VI can be associated with poor prognosis in PTCs. Considering that PMC tumors have a better prognosis, our results are consistent with those reported in the literature. The global prevalence of thyroid carcinoma highlights the importance of early diagnosis and postoperative treatment. The presence of incidental PMC in many patients in the present study is remarkable in terms of the increasing number of PMC cases. The advanced stage of PTC in patients over 45 years of age, male sex, bilaterality, multifocality, VI, and TCI leads to poor prognosis. This highlights the need for more frequent postoperative follow-up in these patients because of the chance recurrence. Although the number of patients with PTC and lymphocytic infiltration in and around the tumor is small, their occurrence in advanced stages contradicts the findings in the literature. We believe that performing FNAB on suspicious lesions measuring less than 1 cm in MNG patients may increase the frequency of PMC detection more.

## Figures and Tables

**Table 1:** Patient characteristics.

Characteristics	Overall, *n* (%)
Age (years)	
>45	428 (60%)
≤45	288 (40%)
Gender	
Female	543 (76%)
Male	173 (24%)
Diagnosis	
ADH	464 (65%)
PMC	134 (19%)
PTC	67 (9%)
FA	23 (3.1%)
FC	18 (2.5%)
OCA	5 (0.7%)
OCC	3 (0.4%)
MC	2 (0.3%)

**Table 2:** Patient and tumor characteristic comparison between PMC and PTC.

Characteristics	PMC (*N* = 134^a^)	PTC (*N* = 67^a^)	*p*-Value
Age (years)			
>45	86 (64%)	37 (55%)	0.219^b^
≤45	48 (36%)	30 (45%)	
Gender			
Female	107 (80%)	46 (69%)	0.079^b^
Male	27 (20%)	21 (31%)	
Localization			
Right	69 (51%)	39 (58%)	0.709^c^
Left	55 (41%)	24 (36%)	
Isthmus	10 (7%)	4 (6%)	
Bilaterality			
Absent	110 (83%)	47 (70%)	0.041^b^
Present	23 (17%)	20 (30%)	
Multifocality			
Absent	114 (85%)	54 (81%)	0.419^b^
Present	20 (15%)	13 (19%)	
Tumor size (cm)	0.40 (0.2–0.6)	2.00 (1.50–3.20)	<0.001^b^
TGF			
Noncapsulated	112 (84%)	27 (40%)	<0.001^b^
Encapsulated	22 (16%)	40 (60%)	
Extra-thyroidal extension			
Absent	127 (95%)	61 (91%)	0.365^c^
Present	7 (5%)	6 (9%)	
Intra-tumoral lymphocyte			
Absent	128 (96%)	61 (91%)	0.220^c^
Present	6 (4%)	6 (9%)	
Nontumoral lymphocyte			
Absent	119 (89%)	59 (88%)	0.875^b^
Present	15 (11%)	8 (12%)	
Intra-tumoral fibrosis			
Absent	122 (91%)	60 (90%)	0.733^b^
Present	12 (9%)	7 (10%)	
Nontumoral fibrosis			
Absent	129 (96%)	61 (91%)	0.185^c^
Present	5 (3%)	6 (9%)	
Disease stage			
pT1	128 (96%)	35 (52%)	<0.001^b^
pT2	1 (0.7%)	22 (33%)	
pT3	5 (3%)	10 (15%)	
TCI			
Absent	122 (91%)	46 (69%)	<0.001^b^
Present	12 (9%)	21 (31%)	
VI			0.012^c^
Absent	134 (100%)	63 (94%)	
Present	0 (0%)	4 (6.0%)	

^a^ *n* (%); median (25–75%).

^b^Pearson's chi-squared test.

^c^Fisher's exact test.

**Table 3:** Comparison between unilateral and bilateral PTC.

	Bilaterality		
Characteristics	Absent, *N* = 47^a^	Present, *N* = 20^a^	*p*-Value
Age (years)			
>45	27 (57%)	10 (50%)	0.575^b^
≤45	20 (43%)	10 (50%)	
Gender			
Female	36 (77%)	10 (50%)	0.032^b^
Male	11 (23%)	10 (50%)	
Tumor size (cm)	2 (1.5–3)	2.55 (1.5–4.12)	0.249^c^
TGF			
Encapsulated	28 (60%)	12 (60%)	0.974^b^
Noncapsulated	19 (40%)	8 (40%)	
Extra-thyroidal extension			
Absent	44 (94%)	17 (85%)	0.353^d^
Present	3 (6%)	3 (15%)	
Intra-tumoral lymphocyte			
Absent	44 (94%)	17 (85%)	0.353^d^
Present	3 (6%)	3 (15%)	
Nontumoral lymphocyte			
Absent	43 (91%)	16 (80%)	0.226^d^
Present	4 (8%)	4 (20%)	
Intra-tumoral fibrosis			
Absent	41 (87%)	19 (95%)	0.665^d^
Present	6 (13%)	1 (5%)	
Nontumoral fibrosis			
Absent	43 (91%)	18 (90%)	>0.999^d^
Present	4 (8%)	2 (10%)	
Disease stage			
pT1	27 (57%)	8 (40%)	0.090^d^
pT2	16 (34%)	6 (30%)	
pT3	4 (8%)	6 (30%)	
TCI			
Absent	31 (66%)	15 (75%)	0.465^b^
Present	16 (34%)	5 (25%)	
VI			
Absent	45 (96%)	18 (90%)	0.577^d^
Present	2 (4%)	2 (10%)	

^a^ *n* (%); median (25–75%).

^b^Pearson's chi-squared test.

^c^Mann–Whitney *U* test.

^d^Fisher's exact test.

**Table 4:** Comparison between unifocal and multifocal PTC.

	Multifocality		
Characteristics	Absent, *N* = 54^a^	Present, *N* = 13^a^	*p*-Value
Age (years)			
>45	32 (59%)	5 (38%)	0.176^b^
≤45	22 (41%)	8 (62%)	
Gender			
Female	39 (72%)	7 (54%)	0.317^c^
Male	15 (28%)	6 (46%)	
Tumor size (cm)	2 (1.50–3.15)	2.50 (1.5–3.5)	0.975^d^
TGF			
Encapsulated	34 (63%)	6 (46%)	0.267^b^
Noncapsulated	20 (37%)	7 (54%)	
Extra-thyroidal extension			
Absent	48 (89%)	13 (100%)	0.588^c^
Present	6 (11%)	0 (0%)	
Intra-tumoral lymphocyte			
Absent	51 (94%)	10 (77%)	0.082^c^
Present	3 (5%)	3 (23%)	
Nontumoral lymphocyte			
Absent	49 (91%)	10 (77%)	0.179^c^
Present	5 (9%)	3 (23%)	
Intra-tumoral fibrosis			
Absent	48 (89%)	12 (92%)	>0.999^c^
Present	6 (11%)	1 (7%)	
Nontumoral fibrosis			
Absent	50 (93%)	11 (85%)	0.329^c^
Present	4 (7%)	2 (15%)	
Disease stage			
pT1	28 (52%)	7 (54%)	>0.999^c^
pT2	18 (33%)	4 (31%)	
pT3	8 (15%)	2 (15%)	
TCI			
Absent	36 (67%)	10 (77%)	0.740^c^
Present	18 (33%)	3 (23%)	
VI			
Absent	50 (93%)	13 (100%)	0.579^c^
Present	4 (7%)	0 (0%)	

^a^ *n* (%); median (25–75%).

^b^Pearson's chi-squared test.

^c^Fisher's exact test.

^d^Mann Whitney U Test.

## Data Availability

Research data supporting this publication are available on request from Istanbul University Cerrahpasa Medical Faculty.
